# Myosin Isoforms and Contractile Properties of Single Fibers of Human Latissimus Dorsi Muscle

**DOI:** 10.1155/2013/249398

**Published:** 2013-07-22

**Authors:** Antonio Paoli, Quirico F. Pacelli, Pasqua Cancellara, Luana Toniolo, Tatiana Moro, Marta Canato, Danilo Miotti, Carlo Reggiani

**Affiliations:** ^1^Department of Biomedical Sciences, University of Padova, Via Marzolo 3, 35031 Padova, Italy; ^2^Salvatore Maugeri Foundation, Pavia, Italy

## Abstract

The aim of our study was to investigate fiber type distribution and contractile characteristics of Latissimus Dorsi muscle (LDM). Samples were collected from 18 young healthy subjects (9 males and 9 females) through percutaneous fine needle muscle biopsy. The results showed a predominance of fast myosin heavy chain isoforms (MyHC) with 42% of MyHC 2A and 25% of MyHC 2X, while MyHC 1 represented only 33%. The unbalance toward fast isoforms was even greater in males (71%) than in females (64%). Fiber type distribution partially reflected MyHC isoform distribution with 28% type 1/slow fibers and 5% hybrid 1/2A fibers, while fast fibers were divided into 30% type 2A, 31% type A/X, 4% type X, and 2% type 1/2X. Type 1/slow fibers were not only less abundant but also smaller in cross-sectional area than fast fibers. During maximal isometric contraction, type 1/slow fibers developed force and tension significantly lower than the two major groups of fast fibers. In conclusion, the predominance of fast fibers and their greater size and strength compared to slow fibers reveal that LDM is a muscle specialized mainly in phasic and powerful activity. Importantly, such specialization is more pronounced in males than in females.

## 1. Introduction

Quadriceps femoris and even more its lateral portion, vastus lateralis (VL), has been the source of most of the present knowledge on “*in vitro*” human muscle fibers characteristics. The choice of VL has been based on the possibility to collect biopsy samples from the bulk of the muscle without the risk of touching important nerve and blood vessels [1]. As additional reasons, the choice has been driven by the possible correlation between muscle characteristics and physical performance as the VL muscle belongs to the group of the leg extensors which can be easily tested (leg extension, jump, etc.) and play an important role in posture and many motor activity (raising from a chair as well as jumping) [2]. In upper limb muscles, biopsy sampling and single fiber characterization have been performed less often, even if upper limb muscles are specifically affected by some muscle diseases (e.g., FSHD or Facio-Scapulo-Humeral muscular Dystrophy [3]) and are specifically involved in some athletic performances and daily activity. Upper limb muscles are in a condition clearly different from lower limb muscles, with respect to postural activity, applied load, and kind of “natural” movement (i.e., walking, running, and jumping for lower limbs versus throwing, climbing, lifting objects for upper limbs). This is reflected in the distinct fiber type composition and, taking MyHC isoforms as markers of fiber type, the proportion of fast MyHC appears definitely higher in upper limbs compared to lower limbs [4].

Data on human single fibers from upper limb muscles are presently available only for the deltoid [5, 6], triceps brachii [6, 7], and biceps brachii [7, 8]. Among muscles acting on the shoulder or glenohumeral joint, Latissimus Dorsi Muscle (LDM) has received great attention as a donor of tissue for cardiomyoplasty since 1987 [9] and, more recently, for mammary reconstruction [10, 11]. For this reason, several studies have examined morphometry and fiber type distribution in LDM [9–19], but no studies on isolated single muscle fibers are available. 

From the physiological point of view, LDM is involved in many daily life activities like pulling an object and in sport movements (rowing, throwing objects, etc.); moreover LDM coactivation during active arm abduction has been reported to have a stabilization action on the shoulder [20]. It is thus surprising that, despite the previously mentioned important functions, studies on LDM fiber distribution and mechanical characteristics are lacking. This prompted us to study LDM at single fiber level in 18 healthy young volunteers. Each single fiber was characterized by its ability to develop isometric force and this functional parameter was related to fiber size or cross-sectional area, to fiber type, based on myosin isoform composition, and to myonuclear density, which seems to be also a determinant of muscle fiber strength [21]. We compared the results with available information on single muscle fibers from VL, taken as representative example of lower limb muscles.

## 2. Materials and Methods

### 2.1. Subjects

Eighteen undergraduate students (9 males and 9 females; age 24.9 ± 5.3 years; 71.15 ± 12.15 kg body weight 176.53 ± 9.35 cm height) of the course of “Human Movement Science” of the University of Padova responded to an invitation to participate in the study. Respondents provided written informed consent to take part in the study and were screened for the presence of diseases or conditions that would place them at risk for adverse responses to exercise. All participants were healthy, nonobese, nonsmokers and were not taking any medications. The study was approved by the Ethical Commission of the Salvatore Maugeri Foundation (Pavia, Italy) where biopsy and medical tests were performed, in accordance with Helsinki's declaration of 1995 as modified in 2000.

### 2.2. Muscle Biopsy and Muscle Fibers Analysis

Biopsy samples were collected from the lateral edge of the LDM at the level of the 5th rib. Tru-cut needles (PRECISA 1410 HS Hospital Service S.p.A. Latina. Italy) with a diameter of 14 G and an insertion cannula length of 100 mm were used. The utilization of a thin try cut needle has been validated in a previous publication [22]. The protocol adopted can be summarized as follows. To localize the spatial coordinates of LDM a strict anatomy palpation protocol was adopted. A careful identification of the biopsy site is extremely important, particularly in large muscles, as LDM, where different regions can play distinct functional roles and therefore possess specific fiber type complement. The tendon of the LDM was identified through palpation as it passes under teres major, forming the posterior wall of the axilla. If extension and medial rotation of the arm is resisted, the tendon can be traced along the medial side of the humerus towards its insertion into the floor of the intertubercular groove of the humerus. Thus, the LDM fascicles can be followed by palpation from under axilla toward waist and iliac crest as far as possible. After locating LDM fascicles the subjects were asked to lie down flat on a bed on the nondominant side with legs on top of each other, knees slightly bent, and the nondominant forearm under the head. The dominant arm was relaxed and bent on the anterior side of the trunk and the skin was marked with a skin pen to define the needle entry point and the needle direction.

To further check the location and the orientation of the LDM fibers ultrasonography was performed by ultrasound probe (Carsi Plus ESAOTE, 7.5–10 MHz probe) first with the muscle relaxed (muscle thickness 0.5–0.8 cm) and then during a maximum voluntary isometric contraction (muscle thickness 0.7–1.8 cm). This allowed the surgeon to confirm the localization of the muscle belly. In a few subjects ultrasonography was performed also during needle insertion. 

After identifying the biopsy location and after local anaesthesia with 2 cc of xylocaine 2% and sterilization with Betadine, the needle was inserted into the muscle. In each subject, three samples were collected from the same location in subsequent insertions of the inner notched rod of the needle. The average yield from each sampling was 4 mg, which corresponds to a cylinder of approximately 0.7–0.9 mm diameter, cross-sectional area of approximately 0.5 mm^2^ and length of about 8 mm (i.e., the full length of the notch on the inner rod). Approximately 500 fiber segments were present in each sample and a good fraction of them reached a length above 0.5 mm, thus allowing their use in single fiber mechanical experiments. The sample to be used for this purpose was immediately immersed in high potassium, high EGTA solution, usually indicated as skinning solution (see below), mixed in equal part with glycerol. Once immersed in this solution, the sample could be stored at −20°C for up to two weeks.

On the day of the experiment, the samples were washed with skinning solution with ATP; single fibers were manually dissected under a stereomicroscope and permeabilized with 1% Triton X-100. Fiber segments of the average length of 0.7 mm were mounted in the setup between the force transducer (model AME-801; SensorOne, Sausalito, CA) and the motor (SI, Heidelberg, Germany) equipped with a displacement transducer by means of light aluminium clips. The fiber segment was immersed in a drop of relaxing solution and, after measuring length, diameters and sarcomere length at 400X magnification was stretched by 20%. It was then transferred into the preactivating solution and finally maximally activated by immersion in the activating solution (pCa 4.6) at 12°C. Isometric force (*F*_0_) was measured in four subsequent maximal activations and average value was calculated. The fiber segment was then removed from the setup and stored in Laemmli solution for electrophoretic determination of MyHC isoform composition.

Skinning, relaxing, preactivating, and activating solutions were prepared as previously described [23]. Their millimolar composition was as follows: (1) skinning solution contained 150 potassium propionate, 5 magnesium acetate, 5 ATP, 5 EGTA, and 5 KH_2_PO_4_; (2) relaxing solution contained 100 KCl, 20 imidazole, 5 MgCl_2_, 5 ATP, and 5 EGTA. Preactivating solution was similar to relaxing solution except that EGTA concentration was reduced to 0.5 mM and 25 mM creatine phosphate and 300 U/mL creatine phosphokinase were added, whereas activating solution was similar to relaxing solution with the addition of 5 mM CaCl_2_, 25 mM creatine phosphate, and 300 U/mL creatine phosphokinase. The pH of all solutions was adjusted to 7.0 at the temperature at which solutions were used (12°C). Protease inhibitors (10 *μ*M E-64 and 40 *μ*M leupeptin) were present in all solutions.

Each fiber was classified by its MyHC isoform composition and characterized by its cross-sectional area (CSA) calculated from three diameters, its isometric force (*F*_0_), and isometric tension (*P*_0_) obtained by normalizing *F*_0_ to CSA.

Proteins for gel electrophoresis were prepared from the remaining of the sample after single fiber dissection and from a sample, specifically collected for this purpose. The tissue samples were solubilized in Laemmli solution (62.5 mM Tris, pH 6.8, 10% glycerol, 2.3% SDS, 5% *β*-mercaptoethanol, with 0.1% E-64 and 0.1% leupeptin as antiproteolytic factors). After heating for 5 min at 80°C, appropriate amounts of the protein suspension were loaded onto polyacrylamide gels (about 1 *μ*g of total protein/lane). For single fibers identification, the whole fiber segment was solubilized in 10 *μ*L of Laemmli solution, and 2-3 *μ*L was loaded onto gels. The separation of MyHC isoforms was achieved on 8% polyacrylamide slab gels with a protocol derived from Talmadge and Roy [24] with some modifications. Slabs 18 cm wide, 16 cm high, and 1 mm thick were used. Electrophoresis was run at 4°C for 24 h, at 70 V for 1 h and 230 V for the remaining time. Three bands were separated in the region of 200 kDa, corresponding (in order of migration from the fastest to the slowest) to MyHC-1, MyHC-2A, and MyHC-2X. Gels were silver stained (Bio-Rad Silver stain plus) for single fiber identification or stained with Coomassie Blue for determination of the relative proportions of the three MyHC isoforms. The relative proportions of MyHC isoforms were determined by the measurement of the brightness-area product B. A. P. (i.e., the product of the area of the band by the average brightness. subtracted local background after black-white inversion) with the accuracy of 600 dpi.

#### 2.2.1. Immunofluorescence

In order to determine the nuclear domain size, from the fiber bundles immersed in skinning solution (see above) single muscle fibers were manually dissected and fixed with 4% paraformaldehyde in PBS for 20 min at room temperature. The fibers were permeabilized with 0.1% Triton X-100 in PBS at room temperature and then incubated in 10% normal goat serum for at least 30 min to block nonspecific antibody binding. Mouse anti-*α*-actinin (clone EA-53 Sigma), a monoclonal antibody, was applied (1:2000) at room temperature in PBS. After 3 washes (10 min each), fluorescent secondary Alexa-568 anti-mouse (Molecular Probes) antibody was incubated for 2 h at room temperature. For visualization of nuclei, single fibers were stained with Hoechst (25 *μ*g/mL; SIGMA) for 10 min. After a final wash in 0.1 M PB, the fibers were mounted in 100% glycerol (Sigma-Aldrich) and covered with a coverslip. The fibers were viewed with a confocal microscope (VICO, Nikon). Serial confocal optical sections (step size: 0.5 *μ*m) were collected by scanning the fiber in only one direction (from top to bottom). The fiber segment volume was reconstructed by adding the volume of the individual sections, each obtained as the product of thickness section (*z*-axis) by surface area (*xy*-axis). The sections were then collapsed on the *z*-axis and the number of nuclei was counted. From nuclei number and volume, the nuclear density (nuclei/10^6^ *μ*m^3^) and the nuclear domain size (*μ*m^3^/nucleus) were obtained. It is worth to observe that the combination of fixation and compression between slides leads to values comparable to other data obtained with similar procedure [25], but lower than those obtained with skinned unfixed fibers [26].

### 2.3. Statistical Analysis

Data are expressed as means and standard error mean unless otherwise indicated. Unpaired *t*-test was used to asset significant differences between male and female. Significance was settled as *P* < 0.05.

## 3. Results

The electrophoretic separation and densitometric determination of MyHC isoform were performed in each of the 18 subjects to delineate the general distribution of fast and slow isoforms. The results showed a clear predominance of fast isoforms with 41.29 ± 2.06% of MyHC 2A and 24.89 ± 1.88 of MyHC 2X, while MyHC 1 represented only 32.84 ± 1.3% (means and SEM. *n* = 18). The predominance of fast isoforms was more pronounced in males. Statistical analysis showed a significant greater percentage of MyHC 1 isoform (*P* < 0.05) and lower percentage of MyHC 2A isoform (*P* < 0.05) fibers in females compared to males, whilst no significant difference was detected for MyHC 2X isoform (see Table 1).

**Figure 1:**
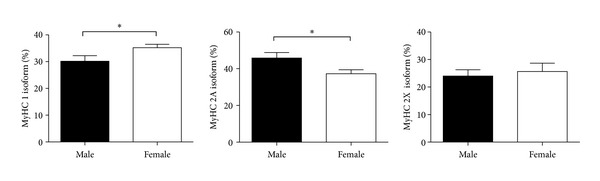
Electrophoretic separation of MyHC isoforms in biopsy samples from LDM. Data are shown as mean and SEM. *N* = 9 for both males and females.

From each subject, at least 15 single muscle fibers were successfully analysed. In each fiber, MyHC isoform composition was determined, cross-sectional area was calculated, and maximal isometric force was measured. The predominance of fast fibers was confirmed as slow or type 1 fibers represented 28%, hybrid 1/2A fibers represented 5%, while fast fibers were divided into 30% type 2A, 31% type A/X, 4% type X, and 2% type 1/2X. Moreover, the proportion of slow fibers was lower in males compared to females (see Figure 1).

**Figure 2:**
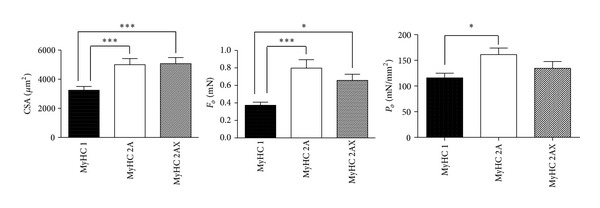
Cross-sectional area isometric force and isometric tension of the three more represented fiber types, classified on the basis of their MyHC (myosin heavy chain) isoform composition. Data are shown as mean and SEM. *P < 0.05; ***P < 0.0005.

The mean cross-sectional area of all muscle fibers analysed was 4654.81 ± 273.08 *μ*m^2^ (mean and SEM. *n* = 286). In the three major groups (see Figure 2), type 1, 2A and 2A/X, each representing approximately 1/3 of the whole population, the mean areas were 3242.59 ± 259.3 *μ*m^2^ (*n* = 69), 4998.63 ± 420.7 *μ*m^2^ (*n* = 74), and 5067.38 ± 418.7 *μ*m^2^ (*n* = 76), for 1, 2A, and 2A/X, respectively. Thus cross-sectional area was significantly greater in fast than in slow fibers (*P* < 0.05).

In the same three groups, as can be seen in Figure 2, the mean isometric force (*F*_0_) was 0.66 ± 0.07 mN for 2A/X fibers, 0.8 ± 0.09 mN for 2A fibers, and 0.37 ± 0.03 mN for type 1 fibers. The isometric specific tension (*P*_0_) was 134.48 ± 13.26 mN/mm^2^ for type 2A/X, 161.3 ± 12.55 mN/mm^2^ for type 2A, and 115.89 ± 17.26 mN/mm^2^ for type 1 fibers; when all fibers were pooled together, *P*_0_ was 142.81 ± 12.55 mN/mm^2^. Thus, slow fibers developed less force and less tension than fast 2A fibers. Interestingly, isometric force was lower in muscle fibers in females compared to males (see Table 1).

In a subset of fibers from 6 randomly selected subjects myonuclear density was calculated as described in Section 2. The results showed that the average myonuclear density was 119.5 ± 3.7 nuclei/10^6^ *μ*m^3^ (mean and SEM. *n* = 78) which corresponds to a volume of the myonuclear domain of 9010 ± 78 *μ*m^3^.

## 4. Discussion

In this study we determined mechanical characteristics of Latissimus Dorsi muscle (LDM) fibers obtained with fine needle biopsy. Only few papers have described LDM fiber type distribution using histochemical or immunohistochemical methods [13, 14, 16, 17, 19, 27, 28] and only one study has also considered the mechanical characteristics [13], although this aspect was not analysed extensively as the main focus was on the regeneration and reinnervation processes.

The emerging picture of the LDM viewed at fiber level is that the muscle is mainly composed of fast fibers, which are not only more abundant but also thicker than slow fibers. Among fast fibers 2A fibers are the majority followed by the group of mixed 2A/X fibers, while pure 2X fibers, although present, are very rare. This fiber type distribution fits well the role of LDM as a muscle with limited postural function, whilst it is commonly used for short, moderate to intense effort [29, 30].

To fully understand the relation between specialization at single fiber level and the functional role of the whole muscles, it is worth to compare the present results obtained in LDM with the characterization of single muscle fibers in VL, a lower limb muscle which combines a pronounced postural duties with the involvement in phasic and powerful activity from raising from the chair to jumping. Among the many studies available published by us [31–34] and other workers [35, 36], we selected for the comparison between LDM and VL the recent paper by Doria et al. [31], as precisely the same methods and the same experimental conditions were applied, and we restricted the comparison to the pretrekking conditions, to avoid confounding effects of a training period. In that study, VL muscle fibers from 7 male young (age 39.4. height 1.72 m, and weight 76 kg) and moderately active subjects were studied.

The myosin isoform expression of VL and LDM appears clearly different as the slow MyHC isoform expression is greater in VL than in LDM: 40.5 ± 0.8% versus 32.84 ± 1.3% (*P* < 0.05). Among the fast isoforms, MyHC 2A proportion is very similar with 41.6 ± 1.6% in VL and 41.29 ± 2.06% in LDM, while MyHC 2X is more abundant in LDM with 24.89 ± 1.88 versus 18.3 ± 1.5% in VL (*P* < 0.05). The fiber type distribution, based on MyHC isoform composition, reflects the proportion of MyHC isoforms with the slow fibers being 35% in VL and only 28% in LDM. The proportion of hybrid fibers is greater in LDM than in VL: 37% versus 30%. A point which deserves a comment is the difference between VL and LDM in cross-sectional area of the slow and fast fibers.  Table 2 shows the values of CSA, *F*_0_, and *P*_0_ obtained in the VL fibers analysed in Doria et al. 2011 [31] in pretrekking conditions, divided in groups on the basis of their MyHC isoform composition and compared with our data of LDM. These data were not present in the paper by Doria and coworkers. As can be seen, in VL the CSA of slow fibers is similar to that of fast fibers, while in LDM slow fibers have a significantly smaller size (see also Figure 2). This is likely related to the different functional tasks of the two muscles: slow fibers which fulfil postural duties are not only more abundant but also thicker in VL than LDM. Importantly, type 1 fibers showed a greater CSA not only in VL but also in other muscles with even broader postural tasks, such as erector spinae muscle. For example, Mannion et al. showed that type 1 fibers had a diameter 62.5 ± 10.0 *μ*m for men and 52.6 ± 4.6 *μ*m for women compared to 59.4 ± 8.4 *μ*m and 41.6 ± 4.7 *μ*m for type 2A [37].

The comparison of the isometric performance in terms of force (*F*_0_) and tension or specific force (*P*_0_) shows that both in VL and LDM the slow fibers are weaker than fast fibers, a feature which can be observed also in other human muscles and in skeletal muscles of other animal species [38]. The lower force of slow fibers seems, thus, an intrinsic functional feature, at least under the experimental conditions adopted to study permeabilized fibers *in vitro*.

The values of nuclear density or of nuclear domain volume which is the reciprocal are worth some comments. The values obtained in LDM are very similar to those obtained by us in a previous study on VL fibers: 119 nuclei/10^6^ *μ*m^3^ versus 122 nuclei/10^6^ *μ*m^3^ [34]. This might indicate that the myoplasm volume controlled by a single myonucleus is not different in upper and lower limb muscles. Two further comments are also useful: (1) the values of nuclear domain volume obtained in this study are lower than those reported in other studies [26] where a different method was adopted. They are comparable to those reported by Mantilla and coworkers [25] who also counted the nuclei and measured the fiber size after the fiber was inserted among two cover slips with a possible effect of compression. (2) The fibers used for nuclear domain determination were not characterized as to the MyHC isoform composition. They were randomly selected and possibly with a different contribution of fast and slow fibers. There is published evidence [26] that slow fibers have a higher nuclear density, or lower nuclear domain volume, than fast fibers. 

The results obtained in this study also provide evidence of a sex-related dimorphism. Some previous papers have also analysed the differences in muscle fiber composition between males and females. For example, Staron and coworkers have analysed sex-related muscle fiber differences in vastus lateralis of young subjects [39]. Fibers were classified as types I, IC, IIC, IIA, IIAB, and IIB and no significant differences between men and women for muscle fiber type distribution were found except for fiber type IC. The percentage distribution of pooled subjects was 41% I, 1% IC, 1% IIC, 31% IIA, 6% IIAB, and 20% IIB. The cross-sectional area of all major fiber types was larger for the men compared to the women, with the exception of type 1 fibers that showed an inverse trend, being larger in women. These data are consistent with those of Yasuda et al. who reported a slight predominance in women compared to men of type I (M: 45.2 ± 10.6%. W: 47.8 ± 9.5%) over type IIa (M: 37.0 ± 6.7%. W: 35.0 ± 3.4%) and type IIx (M: 17.8 ± 8.3%. W: 17.2 ± 10.2%) of vastus lateralis. The comparison of the total area for each fiber type showed that when type 2 fibers are considered together (IIa + IIx), mean fiber area percentage was superimposable between male and female (M: 72.8 ± 3.3% versus W: 68.6 ± 4.6%) whereas women showed a higher type I area compared with men (M: 27.2 ± 3.3% versus W: 31.4 ± 4.6%). Considering the high correlation between the histochemically assessed fractional fiber type area and the electrophoretically assessed MyHC content [40], the data from Yasuda et al. [41] appears to be in agreement with our results on latissimus dorsi: a higher type 1 fiber and a lower type 2 fiber proportion in females compared to males. Further support comes from the data of Martel et al. [42] who showed that in VL type 1 fibers represent 40 ± 3% in males and 47 ± 3% in females, whilst 2A fibers were 31 ± 3% and 32 ± 3%, respectively. Type 2X fibers were 29 ± 3% and 21 ± 4% for males and females. A higher percentage of type 1 fibers in women (67.8 ± 10.5 versus 62.0 ± 9.3) has been found also in Erector spinae muscles counterbalanced by a lower percentage of 2X fibers (4.6 ± 4.7 versus 10.9 ± 6.3) [37]. Thus, the greater abundance of slow fibers in women seems generally supported by published evidence.

In conclusion, the present study provides the first comprehensive analysis of single muscle fibers from human LDM. LDM shares with many upper limb muscles the predominance of fast fibers in causal relation with the low postural duties. Interestingly, the comparison between males and females show that regardless of the postural duties, female muscles are richer in slow fibers.

## Figures and Tables

**Table 1:** Comprehensive table of LDM fibers analysis. Significance between male and female is shown.

	Total (mean ± SEM)	Male (mean ± SEM)	Female (mean ± SEM)	Male versus female
Single fibers characteristics				
CSA *μ*m^2^	4654.81 ± 273.08	6157.12 ± 495.86	3478.01 ± 187.96	*P* < 0.0001
*F*_0_ mN	0.61 ± 0.04	0.85 ± 0.06	0.43 ± 0.03	*P* < 0.0001
*P*_0_ mN/mm^2^	143.39 ± 7.74	160.54 ± 13.48	129.96 ± 8.59	n.s.

Densitometry (% of MyHC)				
MyHC 1	32.84 ± 1.30	30.16 ± 2.06	35.21 ± 1.27	*P* < 0.05
MyHC 2A	41.29 ± 2.06	45.81 ± 3.04	37.28 ± 2.15	*P* < 0.05
MyHC 2X	24.89 ± 1.88	24.03 ± 2.27	25.66 ± 3.04	n.s.

% of number of fibers				
MyHC 1	28.36 ± 0.05	21.09 ± 0.07	35.64 ± 0.05	*P* < 0.0001
MyHC 2A	30.24 ± 0.05	29.58 ± 0.07	30.91 ± 0.08	*P* < 0.0001
MyHC 2X	3.65 ± 0.04	0.00 ± 0.00	7.27 ± 0.07	*P* < 0.0001
MyHC 2A2X	30.97 ± 0.07	43.88 ± 0.10	18.06 ± 0.05	*P* < 0.0001
MyHC 1 2A	5.21 ± 0.02	5.45 ± 0.04	4.97 ± 0.02	*P* < 0.0001
MyHC 1 2A 2X	1.58 ± 0.01	0.00 ± 0.00	3.15 ± 0.02	*P* < 0.0001
MyHC 1 2X	0.00 ± 0.00	0.00 ± 0.00	0.00 ± 0.00	//

**Table 2:** Comparison between present paper and data from previous study performed on vastus lateralis fibers dissected from muscle biopsy samples in the pretrekking conditions from the 7 subjects described in the paper of Doria et al. 2011 [31]. CSA: cross sectional area in *μ*m^2^. *F*_0_ isometric force in mN. *P*_0_ isometric tension in mN/mm^2^.

Fiber type	CSA *μ*m^2^ (mean ± SEM)		*F*_0_ mN (mean ± SEM)		*P*_0_ mN/mm^2^ (mean ± SEM)	
	VL	LD	VL	LD	VL	LD
1	6799 ± 488	3242 ± 259**	0.735 ± 0.076	0.37 ± 0.03**	109.1 ± 9.3	115.89 ± 17.26
2A	6484 ± 653	4998 ± 420	0.808 ± 0.087	0.8 ± 0.09	139.6 ± 14.7	161.3 ± 12.55
2A/2X	5555 ± 663	5067 ± 418	1.029 ± 0.138	0.66 ± 0.07*	190.6 ± 18.1	134.48 ± 13.26*

*P < 0.05; **P < 0.0001.

## References

[1] Tarnopolsky MA, Pearce E, Smith K, Lach B (2011). Suction-modified Bergström muscle biopsy technique: experience with 13,500 procedures. *Muscle and Nerve*.

[2] Paoli A, Marcolin G, Petrone N (2009). The effect of stance width on the electromyographical activity of eight superficial thigh muscles during back squat with different bar loads. *Journal of Strength and Conditioning Research*.

[3] Tawil R, van der Maarel SM (2006). Facioscapulohumeral muscular dystrophy. *Muscle and Nerve*.

[4] Bottinelli R, Reggiani C (2000). Human skeletal muscle fibres: molecular and functional diversity. *Progress in Biophysics and Molecular Biology*.

[5] Helge JW, Overgaard K, Damsgaard R (2006). Repeated prolonged whole-body low-intensity exercise: effects on insulin sensitivity and limb muscle adaptations. *Metabolism*.

[6] O’Sullivan PJ, Gorman GM, Hardiman OM, Farrell MJ, Logan PM (2006). Sonographically guided percutaneous muscle biopsy in diagnosis of neuromuscular disease: a useful alternative to open surgical biopsy. *Journal of Ultrasound in Medicine*.

[7] Harridge SDR, Bottinelli R, Canepari M (1996). Whole-muscle and single-fibre contractile properties and myosin heavy chain isoforms in humans. *Pflugers Archiv European Journal of Physiology*.

[8] Gibala MJ, Interisano SA, Tarnopolsky MA (2000). Myofibrillar disruption following acute concentric and eccentric resistance exercise in strength-trained men. *Canadian Journal of Physiology and Pharmacology*.

[9] Chachques JC, Grandjean PA, Tommasi JJ (1987). Dynamic cardiomyoplasty: a new approach to assist chronic myocardial failure. *Life Support Systems*.

[10] Munhoz AM, Montag E, Fels KW (2005). Outcome analysis of breast-conservation surgery and immediate latissimus dorsi flap reconstruction in patients with T1 to T2 breast cancer. *Plastic and Reconstructive Surgery*.

[11] Chang DW, Youssef A, Cha S, Reece GP (2002). Autologous breast reconstruction with the extended latissimus dorsi flap. *Plastic and Reconstructive Surgery*.

[12] Villa MT, Chang DW (2010). Muscle and omental flaps for chest wall reconstruction. *Thoracic Surgery Clinics*.

[13] Becker MHJ, Wermter TB, Brenner B, Walter GF, Berger A (2000). Comparison of clinical performance, histology and single-fiber contractility in free neurovascular muscle flaps. *Journal of Reconstructive Microsurgery*.

[14] Davidse JHL, van der Veen FH, Lucas CMHB, Penn OCKM, Daemen MJAP, Wellens HJJ (1998). Structural alterations in the latissimus dorsi muscles in three patients more than 2 years after a cardiomyoplasty procedure. *European Heart Journal*.

[15] Hammond DC (2007). Latissimus dorsi flap breast reconstruction. *Clinics in Plastic Surgery*.

[16] Gutierrez PS, Pires WO, Marie SK (2001). Histopathological findings in skeletal muscle used in human dynamic cardiomyoplasty. *The Journal of Pathology*.

[17] Kääriäinen M, Giordano S, Kauhanen S (2011). The significance of latissimus dorsi flap innervation in delayed breast reconstruction: a prospective randomized study-magnetic resonance imaging and histologic findings. *Plastic and Reconstructive Surgery*.

[18] Rifaat M, Amin A, Bassiouny M, Nabawi A, Monib S (2008). The extended latissimus dorsi flap option in autologous breast reconstruction: a report of 14 cases and review of the literature. *Indian Journal of Plastic Surgery*.

[19] Scelsi R, Poggi P, Lo Russo R, De Fabritus F (1993). Effect of preoperative training on Latissimus Dorsi muscle for cardiomyoplasty. A morphometric study. *In Vivo*.

[20] de Witte PB, van der Zwaal P, Visch W (2012). Arm ADductor activation with arm abduction in rotator cuff tear patients vs. healthy controls—design of a new measuring instrument. *Human Movement Science*.

[21] Qaisar R, Renaud G, Morine K, Barton ER, Sweeney HL, Larsson L (2012). Is functional hypertrophy and specific force coupled with the addition of myonuclei at the single muscle fiber level?. *FASEB Journal*.

[22] Paoli A, Pacelli QF, Toniolo L, Miotti D, Reggiani C (2010). Latissimus dorsi fine needle muscle biopsy: a novel and efficient approach to study proximal muscles of upper limbs. *Journal of Surgical Research*.

[23] Toniolo L, Maccatrozzo L, Patruno M (2007). Fiber types in canine muscles: myosin isoform expression and functional characterization. *American Journal of Physiology*.

[24] Talmadge RJ, Roy RR (1993). Electrophoretic separation of rat skeletal muscle myosin heavy-chain isoforms. *Journal of Applied Physiology*.

[25] Mantilla CB, Sill RV, Aravamudan B, Zhan W-Z, Sieck GC (2008). Developmental effects on myonuclear domain size of rat diaphragm fibers. *Journal of Applied Physiology*.

[26] Liu J-X, Höglund A-S, Karlsson P (2009). Myonuclear domain size and myosin isoform expression in muscle fibres from mammals representing a 100 000-fold difference in body size. *Experimental Physiology*.

[27] Johnson MA, Polgar J, Weightman D, Appleton D (1973). Data on the distribution of fibre types in thirty-six human muscles. An autopsy study. *Journal of the Neurological Sciences*.

[28] Kauhanen MS, Salmi AM, von Boguslawsky EK, Leivo IV, Asko-Seljavaara SL (1998). Muscle fiber diameter and muscle type distribution following free microvascular muscle transfers: a prospective study. *Microsurgery*.

[29] Daikoku R, Saito Y (2008). Differences between novice and experienced caregivers in muscle activity and perceived exertion while repositioning bedridden patients. *Journal of Physiological Anthropology*.

[30] Thelen DG, Ashton-Miller JA, Schultz AB (1996). Lumbar muscle activities in rapid three-dimensional pulling tasks. *Spine*.

[31] Doria C, Toniolo L, Verratti V (2011). Improved VO2 uptake kinetics and shift in muscle fiber type in high-altitude trekkers. *Journal of Applied Physiology*.

[32] Pietrangelo T, Mancinelli R, Toniolo L (2009). Effects of local vibrations on skeletal muscle trophism in elderly people: mechanical, cellular, and molecular events. *International Journal of Molecular Medicine*.

[33] Pietrangelo T, Toniolo L, Paoli A (2009). Functional characterization of muscle fibres from patients with chronic fatigue syndrome: case-control study. *International Journal of Immunopathology and Pharmacology*.

[34] Mancinelli R, Kern H, Fulle S (2011). Transcriptional profile of denervated vastus lateralis muscle derived from a patient 8 months after spinal cord injury: a case report. *International Journal of Immunopathology and Pharmacology*.

[35] D’Antona G, Pellegrino MA, Adami R (2003). The effect of ageing and immobilization on structure and function of human skeletal muscle fibres. *Journal of Physiology*.

[36] D’Antona G, Lanfranconi F, Pellegrino MA (2006). Skeletal muscle hypertrophy and structure and function of skeletal muscle fibres in male body builders. *Journal of Physiology*.

[37] Mannion AF, Weber BR, Dvorak J, Grob D, Muntener M (1997). Fibre type characteristics of the lumbar paraspinal muscles in normal healthy subjects and in patients with low back pain. *Journal of Orthopaedic Research*.

[38] Schiaffino S, Reggiani C (2011). Fiber types in Mammalian skeletal muscles. *Physiological Reviews*.

[39] Staron RS, Hagerman FC, Hikida RS (2000). Fiber type composition of the vastus lateralis muscle of young men and women. *Journal of Histochemistry and Cytochemistry*.

[40] Fry AC, Allemeier CA, Staron RS (1994). Correlation between percentage fiber type area and myosin heavy chain content in human skeletal muscle. *European Journal of Applied Physiology and Occupational Physiology*.

[41] Yasuda N, Glover EI, Phillips SM, Isfort RJ, Tarnopolsky MA (2005). Sex-based differences in skeletal muscle function and morphology with short-term limb immobilization. *Journal of Applied Physiology*.

[42] Martel GF, Roth SM, Ivey FM (2006). Age and sex affect human muscle fibre adaptations to heavy-resistance strength training. *Experimental Physiology*.

